# Silymarin Dehydroflavonolignans Chelate Zinc and Partially Inhibit Alcohol Dehydrogenase

**DOI:** 10.3390/nu13124238

**Published:** 2021-11-25

**Authors:** Václav Tvrdý, Marcel Hrubša, Eduard Jirkovský, David Biedermann, Michal Kutý, Kateřina Valentová, Vladimír Křen, Přemysl Mladěnka

**Affiliations:** 1 Department of Pharmacology and Toxicology, Faculty of Pharmacy in Hradec Králové, Charles University, Heyrovského 1203, 500 05 Hradec Králové, Czech Republic; tvrdyvac@faf.cuni.cz (V.T.); hrubsam@faf.cuni.cz (M.H.); JirkovskyE@faf.cuni.cz (E.J.); 2 Laboratory of Biotransformation, Institute of Microbiology of the Czech Academy of Sciences, Vídeňská 1083, 142 20 Prague, Czech Republic; david.biedermann@gmail.com (D.B.); kata.valentova@email.cz (K.V.); kren@biomed.cas.cz (V.K.); 3 Department of Chemistry, Faculty of Science, University of South Bohemia in Ceske Budejovice, Branisovska 1760, 370 05 Ceske Budejovice, Czech Republic; kutym@seznam.cz

**Keywords:** silybin, dehydrosilybin, flavonolignans, alcohol dehydrogenase, zinc, chelation, docking, glutamate dehydrogenase

## Abstract

Silymarin is known for its hepatoprotective effects. Although there is solid evidence for its protective effects against *Amanita phalloides* intoxication, only inconclusive data are available for alcoholic liver damage. Since silymarin flavonolignans have metal-chelating activity, we hypothesized that silymarin may influence alcoholic liver damage by inhibiting zinc-containing alcohol dehydrogenase (ADH). Therefore, we tested the zinc-chelating activity of pure silymarin flavonolignans and their effect on yeast and equine ADH. The most active compounds were also tested on bovine glutamate dehydrogenase, an enzyme blocked by zinc ions. Of the six flavonolignans tested, only 2,3-dehydroderivatives (2,3-dehydrosilybin and 2,3-dehydrosilychristin) significantly chelated zinc ions. Their effect on yeast ADH was modest but stronger than that of the clinically used ADH inhibitor fomepizole. In contrast, fomepizole strongly blocked mammalian (equine) ADH. 2,3-Dehydrosilybin at low micromolar concentrations also partially inhibited this enzyme. These results were confirmed by in silico docking of active dehydroflavonolignans with equine ADH. Glutamate dehydrogenase activity was decreased by zinc ions in a concentration-dependent manner, and this inhibition was abolished by a standard zinc chelating agent. In contrast, 2,3-dehydroflavonolignans blocked the enzyme both in the absence and presence of zinc ions. Therefore, 2,3-dehydrosilybin might have a biologically relevant inhibitory effect on ADH and glutamate dehydrogenase.

## 1. Introduction

**Figure 1:**
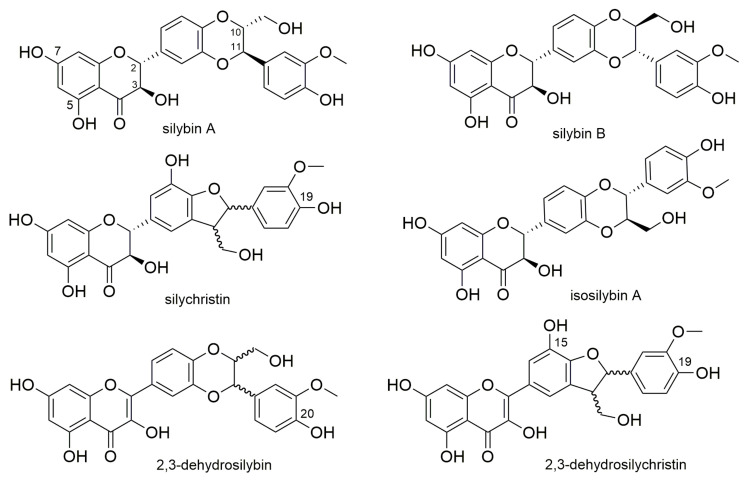
The structures of selected silymarin flavonolignans.

Silymarin, a mixture of polyphenolic compounds extracted from *Silybum marianum* (L.) Gaertn. (Asteraceae) fruits, is well known for its hepatoprotective properties. It is a complex composed mostly of flavonolignans, but it also contains flavonoids (taxifolin, quercetin) and polymeric molecules. The main flavonolignan components contained in silymarin are silybin, isosilybin, silychristin, and silydianin (occurring, except silydianin, as diastereomers A and B), with silybin forming 30–60% of the mixture. Besides flavonolignans and some flavonoids, silymarin also contains ca 30–50% of a polymeric fraction of polyphenols [1]. Silybin, also denoted as silibinin, is the most studied silymarin flavonolignan. Nevertheless, also other flavonolignans including their 2,3-dehydroderivatives (Figure 1) were recently documented to possess interesting pharmacological properties [2,3,4,5].

Thanks to its hepatoprotective properties [6], silymarin is frequently used both as a prescription and an over-the-counter drug, but also in food supplements that claim liver protection. No health claim was, however, approved by the European Food Safety Authority (EFSA) for such supplements. Several studies also suggested that it can protect the liver against excessive alcohol intake [7,8,9]. The organ responsible for the metabolization of more than 90% of ingested alcohol into acetaldehyde and further to acetate is the liver. A number of negative effects of ethanol abuse is in fact caused by its metabolite acetaldehyde, which is mainly generated by alcohol dehydrogenase (ADH), cytochrome P450 (CYP) 2E1, and to a minor extent, catalase [10]. Generally, ADHs are responsible for the majority of alcohol metabolism, however, in chronic drinkers, a significant induction of CYP 2E1 occurs, causing a shift in alcohol metabolism [11].

Since it is well-known that acetaldehyde is toxic to the liver and silymarin can protect against alcohol toxicity, we hypothesize that it can potentially interact with alcohol metabolism. ADH is a zinc-dependent enzyme responsible for the cytosolic oxidation of alcohol to acetaldehyde in the liver and the stomach. It uses nicotinamide dinucleotide (NAD^+^) as a cofactor. In humans, there are several classes of ADH that differ in their structure and kinetics. The most important isoenzymes for ethanol metabolism are ADHs I, II, and IV. These enzymes are dimeric and have a molecular weight of 40 kDa per subunit [12]. They contain two zinc atoms per subunit, which serve different functions in the enzyme. One zinc atom is located in the active site of ADH and is essential for its enzymatic activity [13]. It binds substrates, including ethanol, and participates in an acid-base system for proton removal. The second zinc atom most likely plays a prominent conformational role, probably by stabilizing the tertiary structure of the enzyme [14].

**Figure 2:**
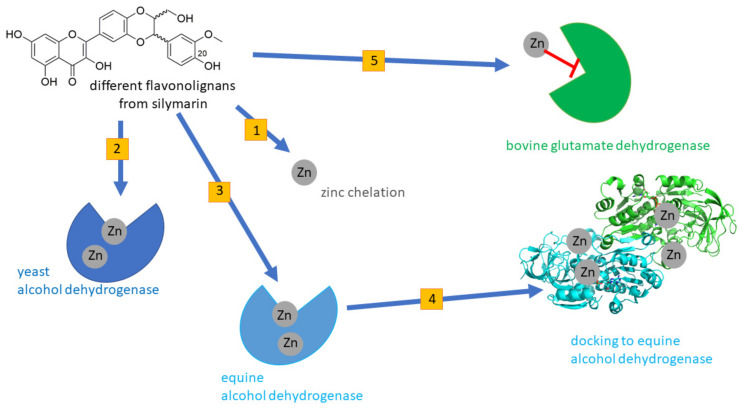
Graphical scheme of the study design. First, flavonolignans were tested for zinc chelation (**1**) and inhibition of yeast alcoholdehydrogenase (**2**). Then active compounds were tested for their interaction with equine alcoholdehydrogenase (**3**) and inhibition was confirmed in a docking study (**4**). Additionally, the interaction of the zinc-chelating flavonolignans with bovine glutamate dehydrogenase (**5**), an enzyme inhibited by zinc ions, was tested.

The ability of silymarin flavonolignans to chelate copper and iron has already been demonstrated by our group [15]. And since metal chelators are often non-selective, we hypothesized that the chelating properties of some of these compounds may result in the inhibition of ADHs by zinc chelation, and hence this could represent an additional factor in their hepatoprotective properties. Although there are some known interspecies differences in the structure of various ADHs [16], leading to different substrate specificity, ethanol reaction kinetics and degree of blockade by known inhibitors, the inhibition of ADH-enzymes by zinc removal could be analogous [17,18]. Hence the aim of this study was to test if (a) flavonolignans from silymarin can chelate zinc ions and (b) if such chelating ability could be biologically relevant. The latter was achieved by testing the selected compounds using both yeast and equine liver ADH. Based on previous studies, the latter enzyme could serve as a model for human liver ADH [17]. Sulfated metabolites of the most potent silymarin components were included in the study to evaluate whether conjugation affects the activity. The graphical scheme of the study design is summarized in Figure 2.

## 2. Materials and Methods

### 2.1. Chemicals

Silymarin was obtained from Liaoning Senrong Pharmaceuticals (Panjin, China, batch No. 120501) and it contained silybin A (9.4%), silybin B (13.8%), silychristin A (9.5%), silychristin B (2.0%), silydianin (4.1%), isosilybin A (3.9%), isosilybin B (1.3%), isosilychristin (0.6%), taxifolin (0.8%), 2,3-dehydrosilybin (0.2%), 2,3-dehydrosilychristin (0.04%), two unknown flavonolignans (both 0.7%) and a polymeric phenolic fraction (53.0%) [19]. Flavonolignans were isolated from silymarin as described previously. Briefly, silybin A + B was obtained by the suspension of silymarin in methanol and filtration yielding solid silybin A + B (49.8% silybin A, 48.0% silybin B). Silybin diastereomers silybin A (99.2%) and silybin B (99.0%) were separated using the diastereomeric enzymatic resolution with immobilized lipase B from *Candida antarctica* (Novozyme 435, Novo-NORDISK, Copenhagen, Denmark) [20]. 2,3-Dehydrosilybin (racemic, 95.0%), 2,3-dehydrosilybin A (97.4%), and 2,3-dehydrosilybin B (98.3%) were synthesized from the respective silybin preparations as described previously [21]. Silychristin (87.1% silychristin A, 9.2% silychristin B) was isolated from silybin-free silymarin by LH-20 chromatography [22]. 2,3-Dehydrosilychristin was prepared by silychristin oxidation [22]. Isosilybin A (99.6%) was obtained from silybin-free silymarin using diastereomeric enzymatic resolution with Novozyme 435 and further purification [20]. 2,3-Dehydrosilybin-20-*O*-sulfate (98.0%), 2,3-dehydrosilybin-7,20-*O*-disulfate (96.0%), 2,3-dehydrosilychristin A 20-*O*-sulfate (98.0%), and isosilybin-20-*O*-sulfate (94.0%) were prepared using the aryl sulfotransferase from *Desulfitobacterium hafniense* [23].

Sodium pyrophosphate (Na_4_P_2_O_7_·10H_2_O), phosphoric acid (purity 85%), monosodium phosphate (NaH_2_PO_4_·H_2_O), disodium phosphate (Na_2_HPO_4_), bovine serum albumin, β-nicotinamide adenine dinucleotide (β-NAD^+^, cat. No. N7381), yeast ADH isolated from *Saccharomyces cerevisiae* (cat. No. A7011), equine ADH (cat. No. A55689), dithizone, zinc chloride, chemicals for buffer preparations, tris(hydroxymethyl)aminomethane (TRIS), hydrochloric acid and sodium hydroxide were purchased from Sigma-Aldrich (St. Louis, MO, USA). *N,N,N′,N′*-Tetrakis(2-pyridylmethyl)ethylenediamine (TPEN) was purchased from Toronto Research Chemicals (North York, ON, Canada). Ultrapure water prepared by Milli-Q RG (Merck Millipore, Burlington, MA, USA) was used throughout this study, while DMSO and ethanol (96%) were purchased from Penta (Prague, Czech Republic).

### 2.2. Zn Chelation In Vitro

To assess the ability of the tested compounds to chelate Zn^2+^ ions, a competitive methodology was used [24]. Briefly, various concentrations of the tested flavonolignan dissolved in DMSO were mixed with an aqueous solution of Zn^2+^ ions (at a final concentration of 10 µM) in different buffers (acetate buffer for pH 4.5, 5.5, and HEPES buffer for pH 6.8, and 7.5). After 2 min of incubation, the indicator dithizone (final concentration 42 µM) was added to quantify the unchelated Zn^2+^ ions spectrophotometrically at 530 nm for pH 4.5 and 540 nm for pH 5.5–7.5. The tested flavonolignans were measured at flavonolignan: Zn^2+^ ions ratios ranging from 1:10 up to 100:1. Preliminary experiments were performed with NEO2MALPHA Synergy 02 (BioTech, Prague, Czech Republic). The subsequent experiments were performed with a Hidex Sense multiplate reader (Hidex, Turku, Finland). The limited solubility of 2,3-dehydrosilybin was observed at higher ratios at pH 4.5, 5.5, and 6.8, and due to this fact, the highest ratio of 100:1 was not included in this case. Flavonolignans without chelation activity at pH 7.5 and 6.8 were not tested at lower pH conditions.

### 2.3. Alcohol Dehydrogenase

#### 2.3.1. Yeast ADH

**Figure 3:**
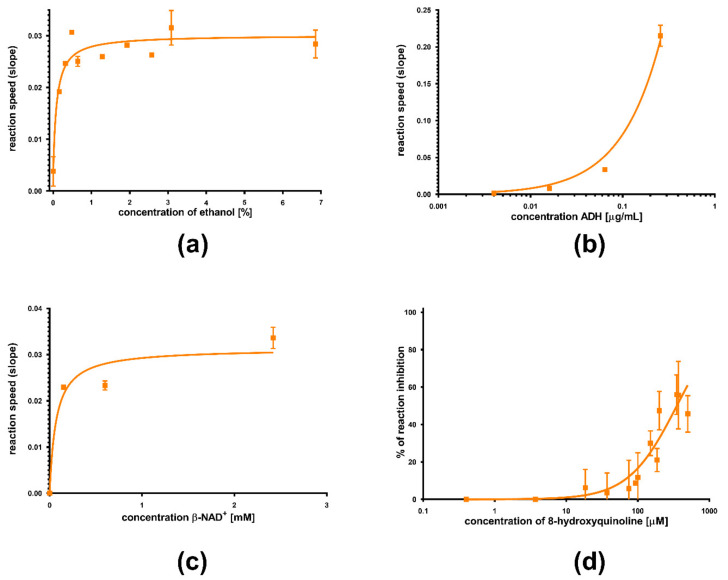
Different experimetal setups in the implementation of the method with yeast ADH. The dependence of the reaction rate on (**a**) ethanol concentration (the final concentration of ADH was 0.64 µg/mL, i.e., 0.02 IU/mL and of β-NAD^+^ was 2.4 mM), (**b**) enzyme amount (the final concentration of ethanol was 7% and of β-NAD^+^ was 2.4 mM), and (**c**) cofactor concentration (the final concentration of ADH was 0.64 µg/mL and that of ethanol was 7%) was tested. Finally, the ability of 8-hydroxyquinoline to inhibit the enzyme was tested (**d**).

The method used for yeast ADH was adapted from Kagi & Vallee [13] with minor modifications. Initially, various experimental conditions (different concentrations of ethanol and β-NAD^+^, amount of enzyme and concentration-dependent inhibition by 8-hydroxyquinoline, a compound specified by the manufacturer as an inhibitor) were performed to optimize the protocol (Figure 3a–d). The tested compound dissolved in DMSO was incubated for 3 min with yeast ADH at a final concentration of 0.064 μg/mL (0.02 IU/mL) in wells of a 96-well plate with sodium phosphate buffer (SPB) of pH 8.8. The enzyme solution added to the wells was prepared by diluting a stock solution of yeast ADH (1 mg/mL) with the enzyme diluent (10 mM SPB of pH 7.5 with 1 mg/mL bovine serum albumin). Ethanol was added as a substrate for the reaction. Its final concentration was 7% (*v*/*v*). Afterward, the conversion of ethanol was initiated by adding β-NAD^+^ (final concentration 2.4 mM). An increase in the measured absorbance corresponding to the reduction of β-NAD^+^ was used to determine the kinetics of the reaction. Measurement was performed using a Tecan 200 Pro Reader at 340 nm for 6 min. The flavonolignan solvent (DMSO) with the enzyme solution was used as the positive control; DMSO and enzyme diluent were used as the blank. Finally, a clinically used inhibitor of ADH fomepizole (4-methylpyrazole), as well as the standard zinc-chelator TPEN were used for comparison.

#### 2.3.2. Equine ADH

The ability of the tested compounds to inhibit recombinant equine ADH was determined in a similar manner as for yeast ADH (Section 2.3.1). The tested compound dissolved in DMSO was incubated with recombinant equine ADH at a final concentration 0.32 mg/mL (0.16 IU/mL) in wells containing 100 mM TRIS buffer of pH 9.5 and 7% (*v*/*v*) of ethanol. Afterwards, the conversion of ethanol was initiated by adding β-NAD^+^ (final concentration 2.4 mM). The time-dependent changes in absorbance at 340 nm were again measured similarly as with the yeast enzyme.

### 2.4. Bovine Glutamate Dehydrogenase

**Figure 4:**
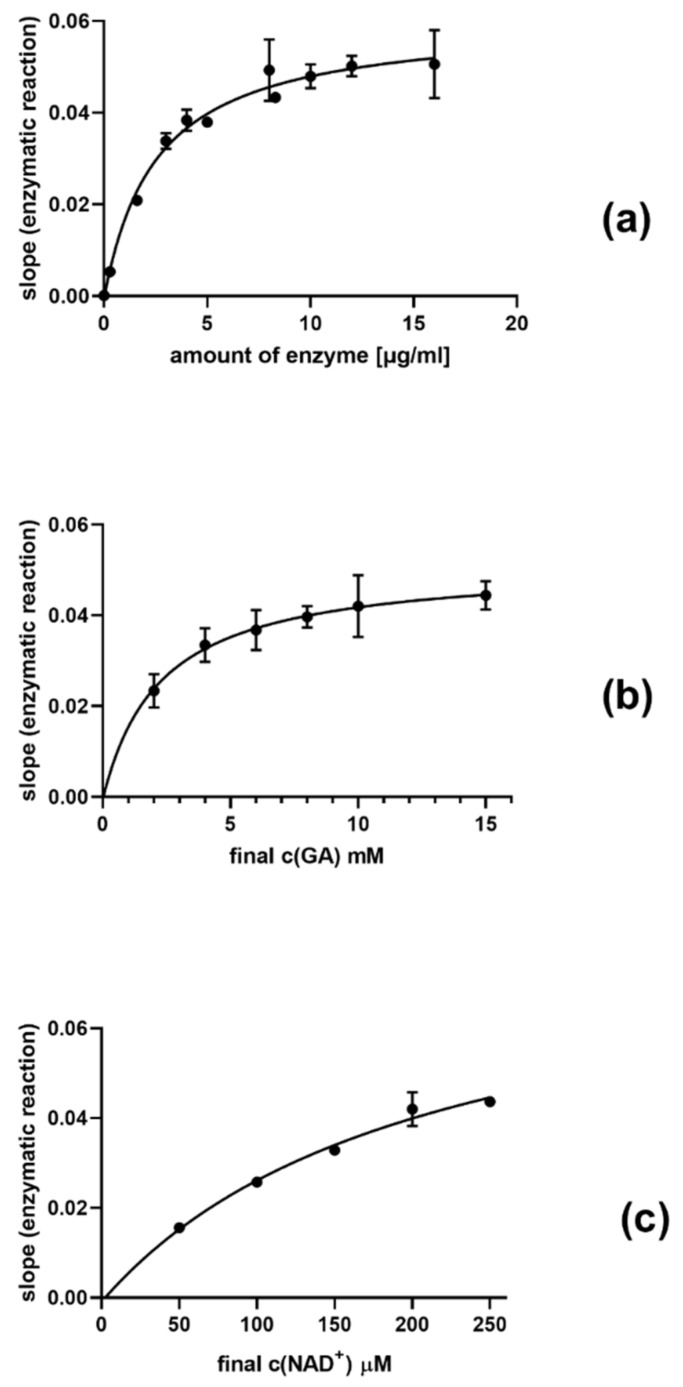
Different experimental setups while performing the implementation of the methodology with bovine glutamate dehydrogenase. The dependence of the reaction rate on (**a**) the amount of the enzyme (the final concentration of glumatate was 10 mM and that of NAD^+^ 200 μM), (**b**) the substrate (glutamate, GA) concentration (the final concentration of the enzyme was 10 μg/mL and that of NAD^+^ 200 μM), (**c**) and co-factor (NAD^+^) concentration (the final concentration of the enzyme was again 10 μg/mL and that of glutamate 10 mM) was tested.

The tested compounds dissolved in DMSO (max 5% DMSO) were incubated with bovine glutamate dehydrogenase type I (Sigma-Aldrich, St. Louis, MO, USA, GDH, final concentration 10 μg/mL) and 5.5 mM glutamate for 3 min. Afterwards, 10 μL of ultrapure water or an aqueous solution of zinc chloride in the final concentration of 150 μM was added. The reaction was initiated by β-NAD^+^ in a final concentration of 185 μM and monitored spectroscopically at 340 nm for 3 min. Negative control contained DMSO and ultrapure water, whereas zinc chloride served as the positive control. All experiments were performed in 96 well microplates. The concentrations of reagents written above were selected according to optimization experiments (Figure 4).

### 2.5. Molecular Docking In Silico

To confirm the accessibility of flavonolignans to the active site of alcohol dehydrogenase, the molecular docking study was performed on the experimental crystal structure of ADH from horse liver in complex with NADH, which was downloaded from the Protein Data Bank (www.rscb.org; PDB ID: 4xd2, released 14 January 2015). Calculations of the electrostatic potential molecular surface of ADH were performed using the freely available macromolecular electrostatic calculation program Adaptive Poisson-Boltzmann Solver (APBS, included as a plugin in PyMOL).

Computational docking studies were performed using AutoDock Vina v.1.1.2 (La Jolla, CA, USA) [25], implemented in UCSF Chimera 1.3.1 [26]. The crystal structure of the protein was docked with 2,3-dehydrosilychristin A, 2,3-dehydrosilybin A and 2,3-dehydrosilybin B. Before the docking process, the hydrogen atoms and the proper charges were automatically added and assigned to the ligands and the target ADH. Then, the docking study was performed over the entire dimeric protein and all selected ligands were docked into the suspected active site where the NAD^+^/NADH coenzyme was present or absent. Docking searches were performed with depletion of 8, 10 modes and energy ranges of 3 kcal·mol^−1^. Searches were carried out over the entire molecule allowing ligands to be flexible. The ViewDock tool in the Chimera package was used to facilitate interactive analysis of the enzyme-ligand docking results. Different poses of the ligand were considered individually in the context of the binding site and ranked according to the energy score, which follows the X-Score [27] scoring function for free binding energies for protein-ligand complexes with known 3D structures. The results were also examined and visualized in PyMOL [28].

### 2.6. Calculations of Experimental Results

Chelation was expressed as concentration-dependent metal chelation curves corresponding to the equation y = maximal chelation/(1 + 10^((^^logEC50−x)×slope)^) where y is the percentage of zinc chelation and x is the decadic logarithm of flavonolignan/Zn^2+^ concentration ratio. Each measurement was performed 3–6 times. A detailed calculation was reported in our previous article [24].

Enzymatic activity was calculated using the increase in absorbance with the subtraction of corresponding blanks. Inhibition was expressed using the equation y = 1 − (slope_compound_/slope_control_). Each measurement was performed 4 times, blanks were made in duplicates.

### 2.7. Statistical Analysis

Data are shown as means ±SD. 95% confidence (prediction) intervals were used for the statistical comparison of zinc chelation and effect on equine ADH. For yeast ADH, comparison was performed at one concentration using ANOVA followed by Dunnett’s multiple comparisons test. All statistical analyses were carried out using GraphPad Prism 8 for Windows (GraphPad Software, San Diego, CA, USA).

## 3. Results

**Figure 5:**
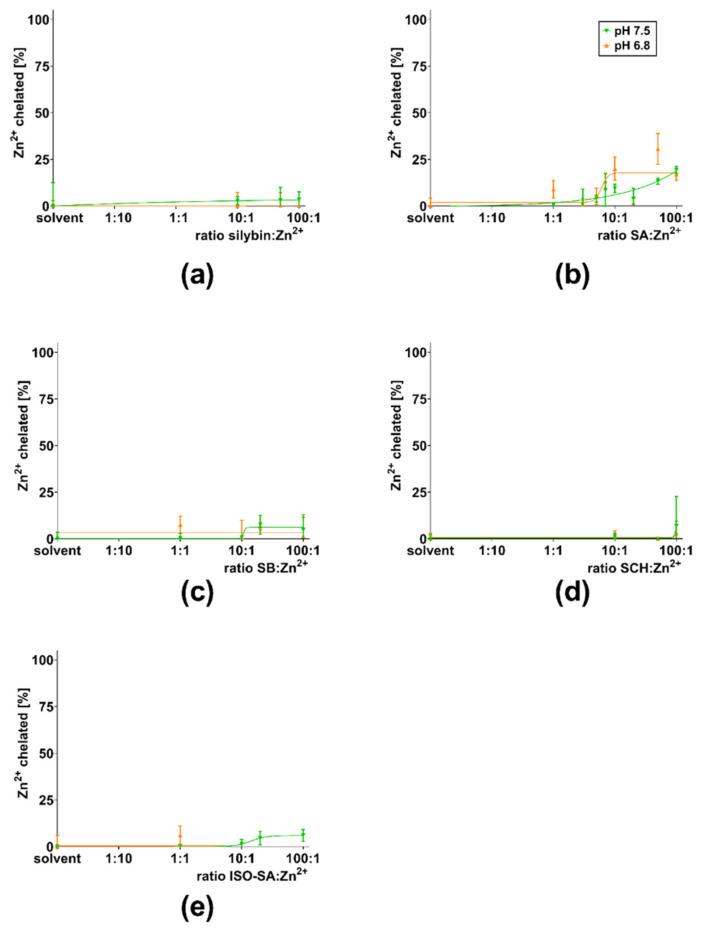
Summarized data of compounds with no or little ability to chelate Zn^2+^ ions; (**a**) silybin A + B, (**b**) silybin A (SA), (**c**) silybin B (SB), (**d**) silychristin (SCH), and (**e**) isosilybin A (ISO-SA) at pH 6.8 and 7.5. The effect at lower pH was not studied due to low activity at these compounds at higher pH conditions.

**Figure 6:**
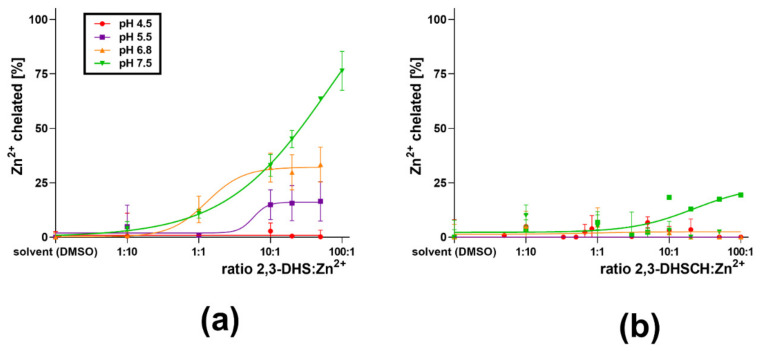
Zn^2+^ chelation activity of (**a**) 2,3-dehydrosilybin (DHS) and (**b**) 2,3-dehydrosilychristin (DHSCH) at different pH levels.

**Figure 7:**
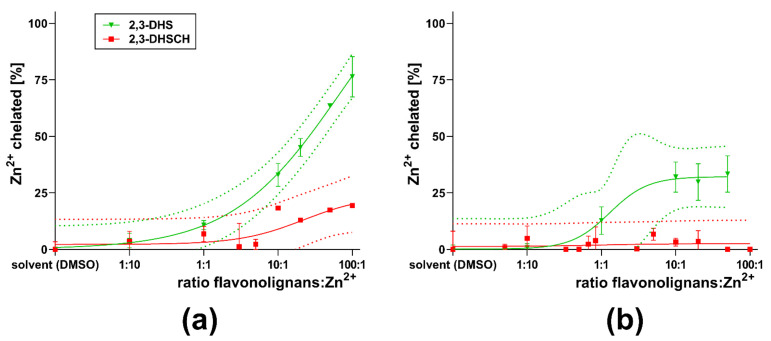
Comparison of ability to chelate Zn^2+^ ions by 2,3-dehydrosilybin (2,3-DHS) and 2,3-dehydrosilychristin (2,3-DHSCH) at (**a**) pH 7.5 and (**b**) 6.8.

First, flavonolignans were tested for their ability to chelate zinc. Flavonolignans saturated at C-2–C-3 (silybin A + B, silybin A, silybin B, isosilybin A, and silychristin) exhibited little or no activity (Figure 5). Only 2,3-dehydro-derivatives of flavonolignans (2,3-dehydrosilybin and 2,3-dehydrosilychristin) exhibited an ability to bind Zn^2+^ ions. The chelating ability of 2,3-dehydrosilybin decreased as the conditions became more acidic, and no chelation at all was observed at pH 4.5 (Figure 6a). For 2,3-dehydrosilychristin, some Zn^2+^ ion-binding properties were observed only at pH 7.5, while no chelation activity was detected under more acidic conditions (Figure 6b). A comparison of confidence intervals confirmed that 2,3-dehydrosilybin was a significantly stronger chelating agent than 2,3-dehydrosilychristin at pH 7.5 and 6.8, while their zinc chelating activities were statistically comparable at lower pH levels (Figure 7).

**Figure 8:**
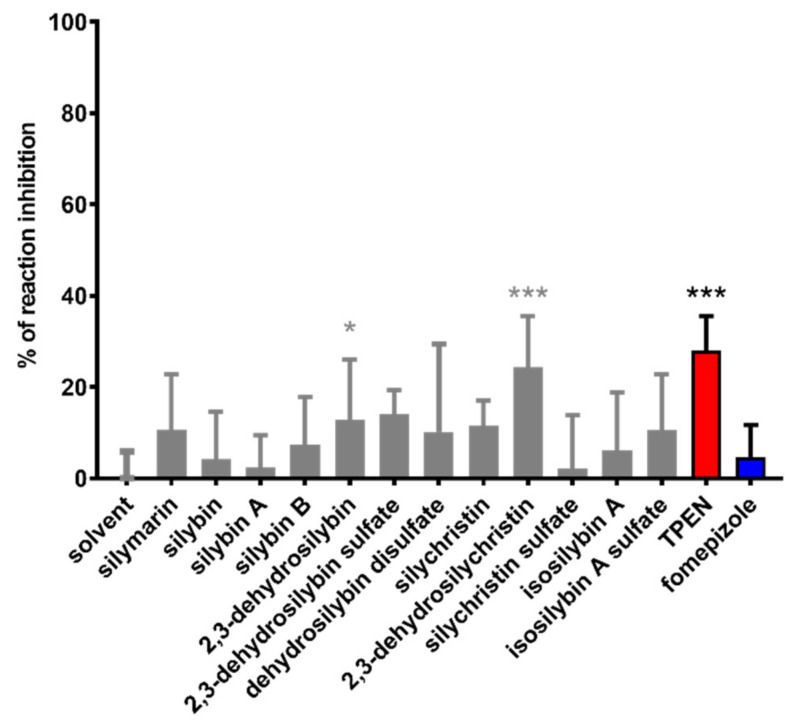
Comparison of all tested flavonolignans and standards in inhibition of yeast ADH. All compounds were tested at a concentration of 100 µM, except for the inactive 2,3-dehydrosilybin disulfate, which was tested at a concentration of 150 µM. * *p* < 0.05 vs. solvent, *** *p* < 0.001 vs. solvent.

**Figure 9:**
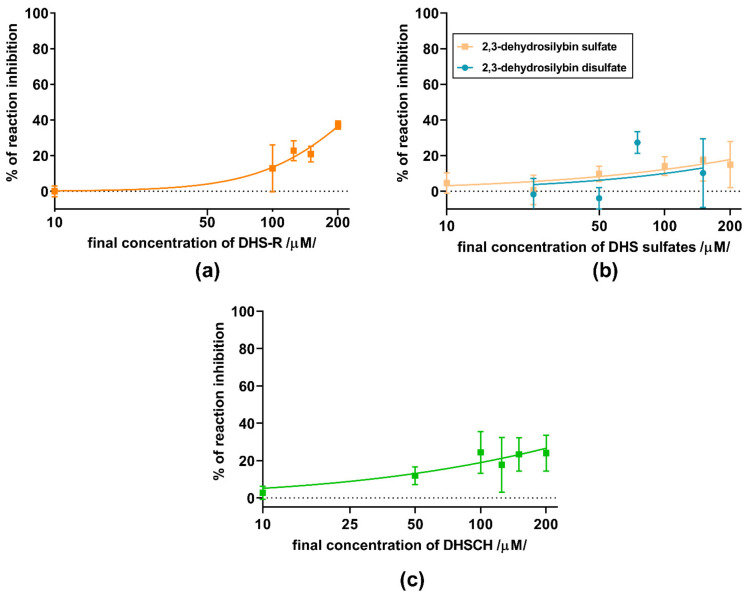
Inhibition of yeast ADH by 2,3-dehydroflavonolignans; (**a**) 2,3-dehydrosilybin (DHS-R), (**b**) 2,3-dehydrosilybin sulfates (mono and disulfate) and (**c**) 2,3-dehydrosilychrystin (DHSCH).

**Figure 10:**
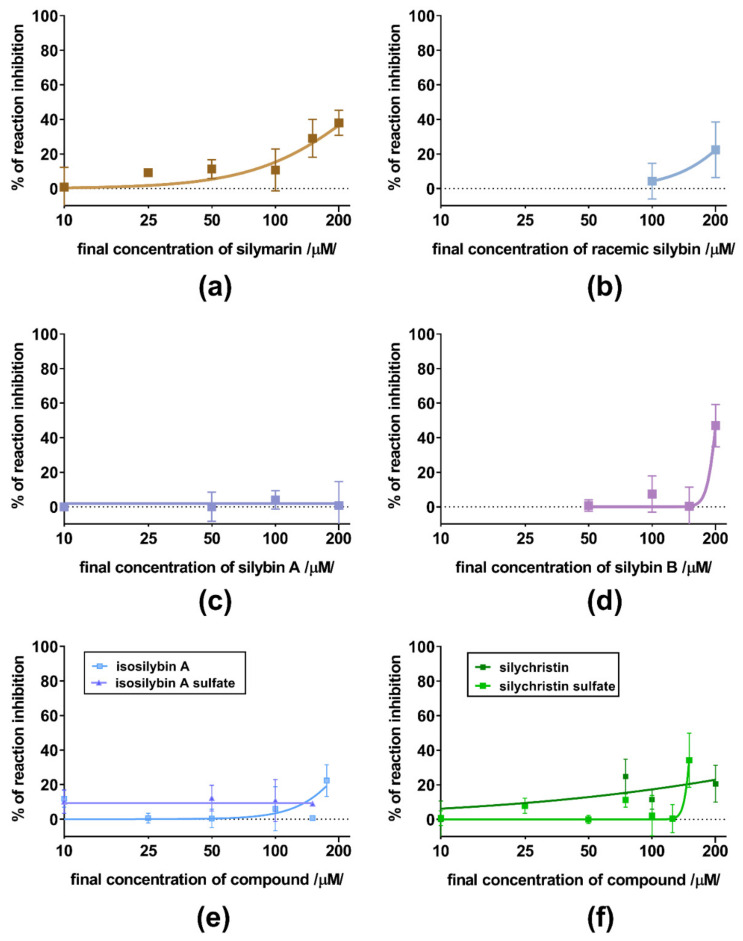
The concentration-dependent inhibition of yeast ADH by weakly active or inactive silymarin flavonolignans; (**a**) silymarin, (**b**) silybin racemate, (**c**) silybin A, (**d**) silybin B, (**e**) isosilybin A and isosilybin A sulfate, and (**f**) silychristin and silychristin sulfate.

**Figure 11:**
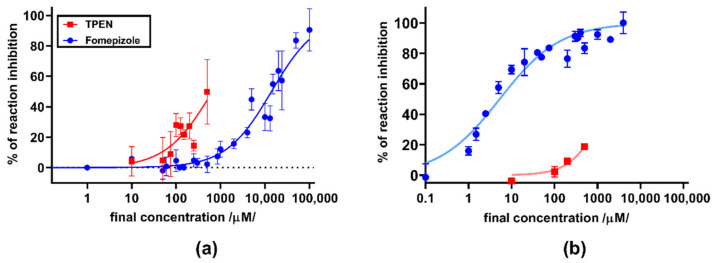
Inhibition of (**a**) yeast and (**b**) equine ADH by fomepizole and TPEN.

Subsequent assays on yeast ADH were performed with a series of 11 flavonolignans, also including silymarin and sulfated metabolites of 2,3-dehydrosilybin (20-*O*-sufate, and 7,20-*O*-disulfate) and 2,3-dehydrosilychristin (20-*O*-sulfate). Hence, non-chelating flavonolignans and metabolites were also included to test whether there is a relationship between zinc chelation and ADH inhibition. In general, all compounds exhibited only weak inhibitory activity at 100 μM (Figure 8), with maximal inhibition not exceeding 35% of uninhibited reaction at concentrations ranging from 10 to 200 μM. Dose-dependent inhibition was observed mainly for silymarin, 2,3-dehydroderivatives (Figure 9 and Figure 10), the standard inhibitor fomepizole and zinc chelator TPEN (Figure 11a). Only two tested compounds (2,3-dehydrosilybin and 2,3-dehydrosilychristin) and TPEN exhibited significant activity at 100 μM compared to the control, i.e., the solvent (Figure 8 and Figure 9). The standard ADH inhibitor fomepizole almost completely inhibited ethanol metabolism by yeast ADH. However, the concentrations required for the inhibition were three orders of magnitude higher than with the others and the IC_50_ value was 13.99 mM (Figure 11a). The inhibitory activity of the standard zinc chelator TPEN was more pronounced than that of fomepizole (IC_50_ 654 μM).

**Figure 12:**
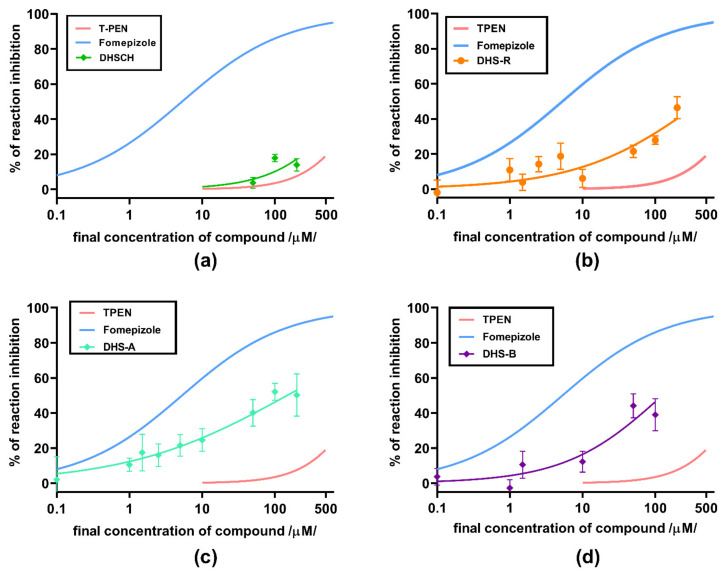
Inhibition of equine ADH by active flavonolignans: (**a**) 2,3-dehydrosilychristin (DHSCH); (**b**) racemic 2,3-dehydrosilybin (DHS); (**c**) 2,3-dehydrosilybin A (DHS-A); and (**d**) 2,3-dehydrosilybin B (DHS-B). Inhibitory curves of fomepizole and TPEN are depicted for comparison. Compounds dissolved in DMSO were mixed with the enzyme (in a final concentration of 0.32 mg/mL/0.16 IU/mL/) and ethanol 7% (*v*/*v*). NAD+ was added at a final concentration of 2.4 mM to initiate the reaction.

In the next step, two of the most active flavonolignans were tested on the equine enzyme together with the two standards fomepizole and TPEN (Figure 11b and Figure 12). Here, fomepizole was much more active compared to yeast ADH, with an IC_50_ of 5.25 µM. The activity of TPEN was similar to that observed with the yeast ADH (Figure 11a,b, respectively). 2,3-Dehydrosilychristin had approximately the same activity as TPEN, whereas 2,3-dehydrosilybin was more active (Figure 12). To test whether stereochemistry has some effect, we compared racemic 2,3-dehydrosilybin with the individual stereoisomers 2,3-dehydrosilybin A and 2,3-dehydrosilybin B. In this case, no significant difference in activity was observed (Figure 12b–d). Besides this, a concentration of 10 µM of 2,3-dehydrosilybin A blocked approximately 20% of the activity of the enzyme, and its inhibitory activity improved with increasing concentrations.

**Figure 13:**
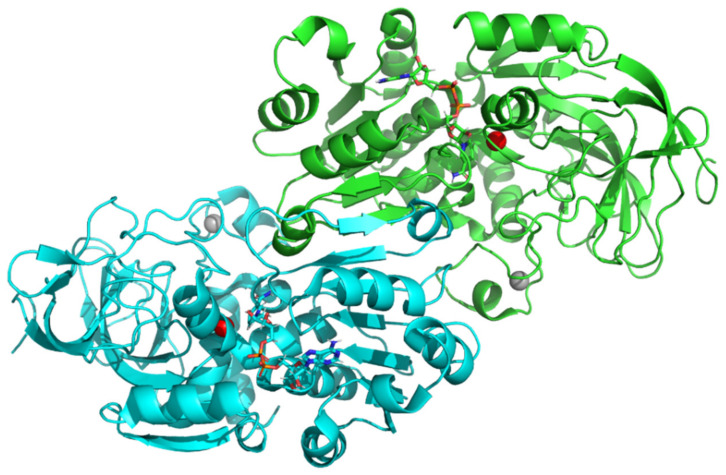
The X-ray ribbon model of the mammalian (equine) ADH dimer (green and cyan chains)—downloaded from the Protein Databank (PDB ID 4xd2), including two stick models of the coenzyme nicotinamide adenine dinucleotide (NAD^+^/NADH) and four spheres of Zn (red in the active site and grey in the peripheral parts of protein).

**Figure 14:**
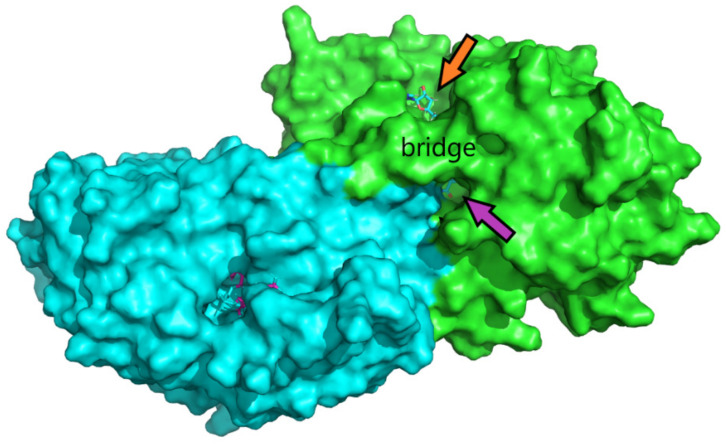
The surface model of the dimeric structure of ADH (green and cyan chains) visualizes two main tunnels (in the monomers of the dimeric structure of ADH) leading to the active site of ADH. The “bridge part” (consisting of residues Thr56, Leu57, Val58, Pro296, Asp297, and Ser298) above the large cavity forms a tunnel. The orange and purple arrows show the entrance for the coenzyme NAD^+^/NADH (stick model) and substrate, respectively. NAD^+^/NADH is visible from both tunnel entrances; the nicotinamide moiety interacts with the substrate entrance. Both chains are involved in the formation of the substrate tunnel.

**Figure 15:**
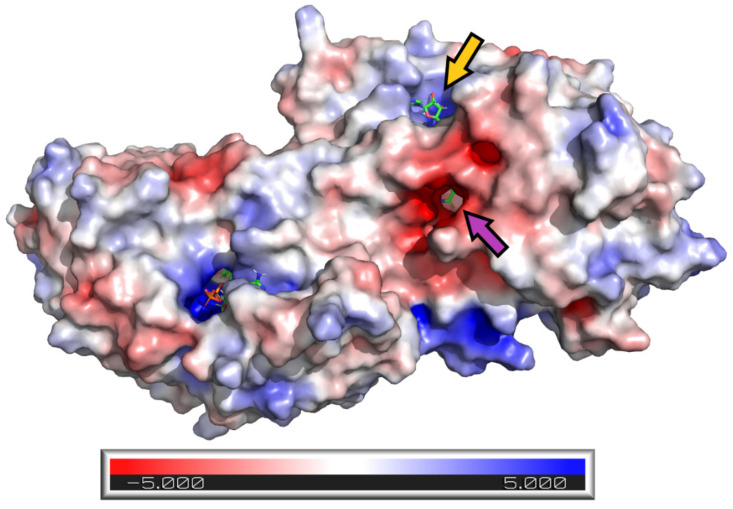
Adaptive Poisson-Boltzmann Solver (APBS) calculations of an electrostatic potential molecular surface area of ADH show the results as a (−5, 5) red–white–blue color-coded electrostatic surface area (in units of kJ/mol/e). The blue region within the NAD^+^/NADH tunnel represents a positive potential attracting the negative phosphate moiety of NAD^+^/NADH. The red region within the substrate tunnel represents a negative potential that attracts the hydroxy group of the substrate (alcohol) or inhibitor (flavonolignans) into the active site.

To confirm the interaction of active dehydroflavonolignans with ADH, the X-ray structure of the dimeric form of equine ADH was visualized (Figure 13 and Figure 14) and examined (Figure 15) to find suitable regions for molecular docking. The surface model reveals tunnels leading to the active site of ADH. The “bridge” region (consisting of residues Thr56, Leu57, Val58, Pro296, Asp297, and Ser298) over the large cavity forms two interconnected tunnels, the first for the coenzyme NAD^+^/NADH and the second for the substrate. The large NAD^+^/NADH molecule extends to both tunnel entrances, the nicotinamide moiety interacts with the substrate tunnel. As can be seen from the surface model (Figure 14), both dimer chains are involved in the formation of the substrate tunnel. Therefore, the entire dimeric structure was included in the molecular docking study.

APBS calculations of an electrostatic potential molecular surface of ADH (Figure 15) show a color-coded electrostatic surface that facilitates the understanding of the nature of intermolecular noncovalent interactions in the examined tunnel. According to these APBS calculations, the tunnel consists of two different electrostatic potential areas. The positive potential area attracts negatively charged diphosphate moiety of the coenzyme NAD^+^/NADH, and the negative potential region (representing the substrate entrance) attracts the hydroxy group of the substrate (alcohol) or inhibitor (a dehydroflavonolignan) to the active site. Thus, in addition to the surface model, the APBS calculations also reveal two important tunnel areas, one is responsible for coenzyme binding and the second for substrate binding.

**Figure 16:**
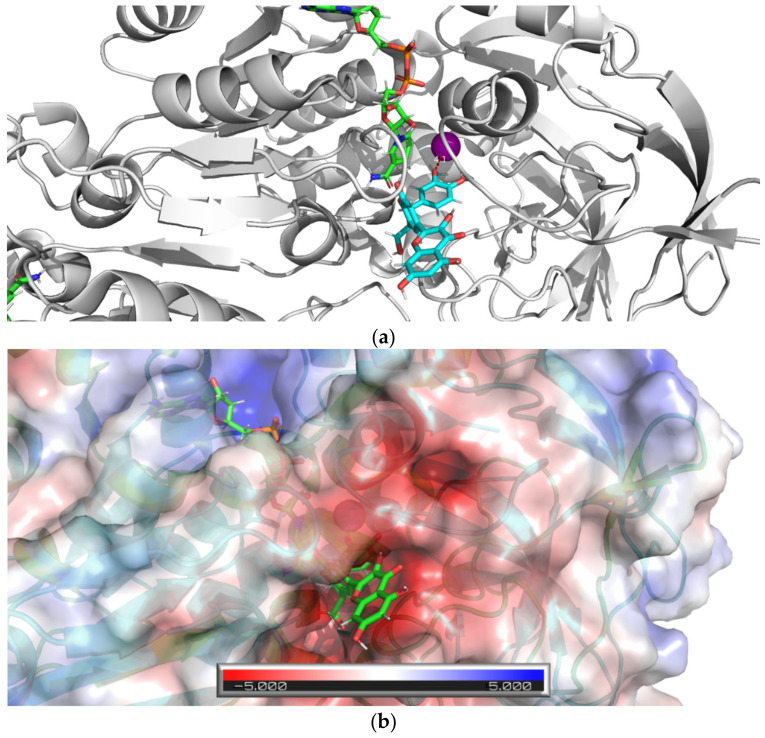
Docking of 2,3-dehydrosilychristin A into equine ADH. The figure shows the most favorable binding mode (energy score −7.1 kcal/mol) of 2,3-dehydrosilychristin A docked into the active site of ADH: (**a**) Ribbon model of ADH and stick model of ligand and NAD, the ligand (the oxygen of the methoxy group) is 4.1 Å far from the catalytic zinc position (magenta sphere); (**b**) Ribbon and partially transparent APBS surface model of ADH together with stick model of ligand (bottom) and NAD (top).

**Figure 17:**
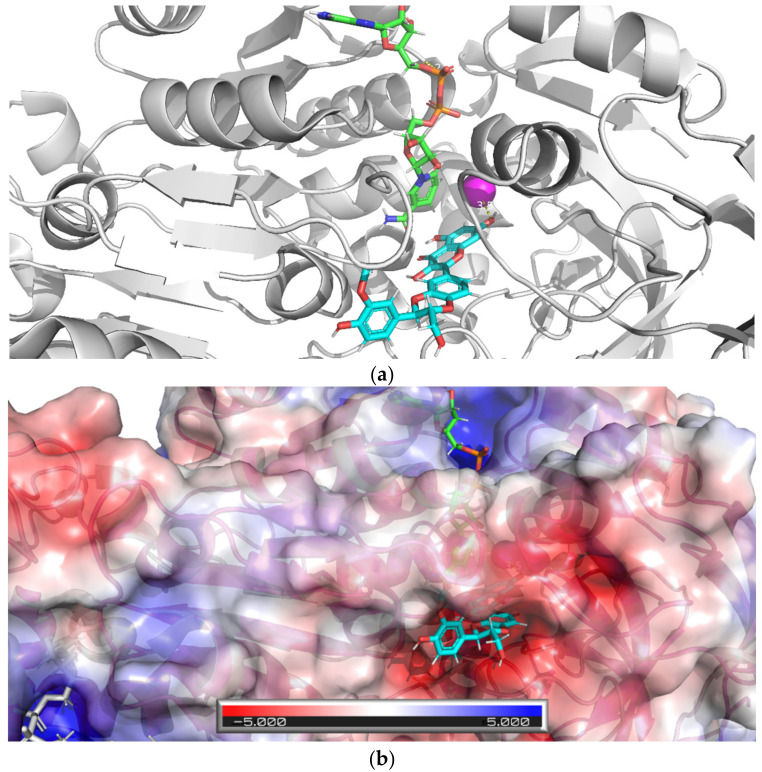
The docking of 2,3-dehydrosilybin A with equine ADH. The figure shows the favorable binding mode (energy score −2.7 kcal/mol) of the ligand 2,3-dehydrosilybin A docked into the ADH active site: (**a**) The ribbon model of ADH and the stick model of the ligand and NAD^+^/NADH; the ligand (oxygen of hydroxy group) is 3.5 Å far from the catalytic zinc position (magenta sphere); (**b**) Ribbon and partially transparent APBS surface model of ADH together with the stick model of the ligand (bottom) and NAD^+^/NADH (top).

**Figure 18:**
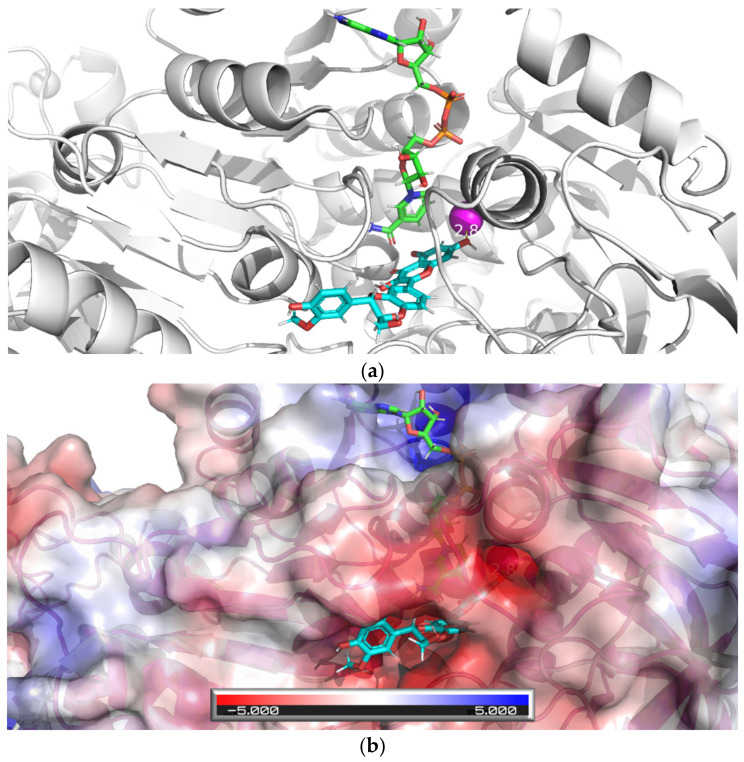
Docking of 2,3-dehydrosilybin A with equine ADH. The figure shows the most favorable binding mode (energy score −2.0 kcal/mol) of 2,3-dehydrosilybin B docked into the ADH active site: (**a**) Ribbon model of ADH and stick model of ligand and NAD, the ligand (oxygen of hydroxy group) is 2.8 Å far from the catalytic zinc position (magenta sphere); (**b**) Ribbon and partly transparent APBS surface model of ADH together with stick model of ligand (bottom) and NAD (top).

The results of the docking studies for two active dehydroflavonolignans including both 2,3-dehydrosilybin diastereoisomers are summarized graphically (Figure 16, Figure 17 and Figure 18). For each ligand, only one pose was selected according to the lowest binding energy. The most favorable binding mode (energy score −7.1 kcal/mol) of 2,3-dehydrosilychristin A was located near the catalytic zinc with the shortest distance of 4.1 Å from the oxygen of the methoxy group (Figure 16). Only one binding mode (energy score −2.7 kcal/mol) of 2,3-dehydrosilybin A was found near the catalytic zinc with the shortest distance of 3.5 Å from the oxygen of the hydroxy group (Figure 17). The most favorable binding mode (energy score −2.0 kcal/mol) of 2,3-dehydrosilybin B was found near the catalytic zinc with the shortest distance of 2.8 Å from the oxygen of hydroxy group (Figure 18). Hence, these docking results support our conclusions from the experimental part of this work.

**Figure 19:**
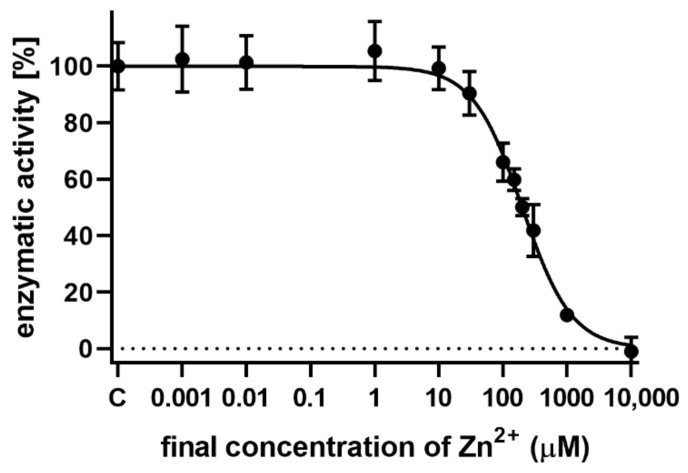
The inhibitory effect of zinc on bovine glutamate dehydrogenase. The final concentration of glutamate was 5 mM and NAD^+^ was added at a final concentration of 185 μM to start the reaction.

**Figure 20:**
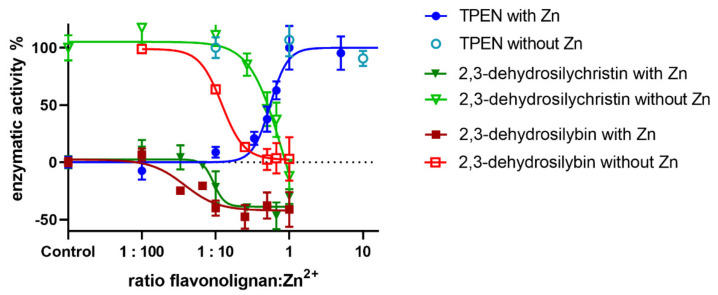
The effect of the tested compounds on bovine glutamate dehydrogenase in the presence or absence of zinc ions. The final concentration of zinc ions was 150 µM. Tested compounds were dissolved in DMSO, final concentration of GDH was 10 μg/mL and NAD^+^ was added at a final concentration of 185 μM to initiate the reaction. Glutamate concentration remained the same as above (5 mM).

**Figure 21:**
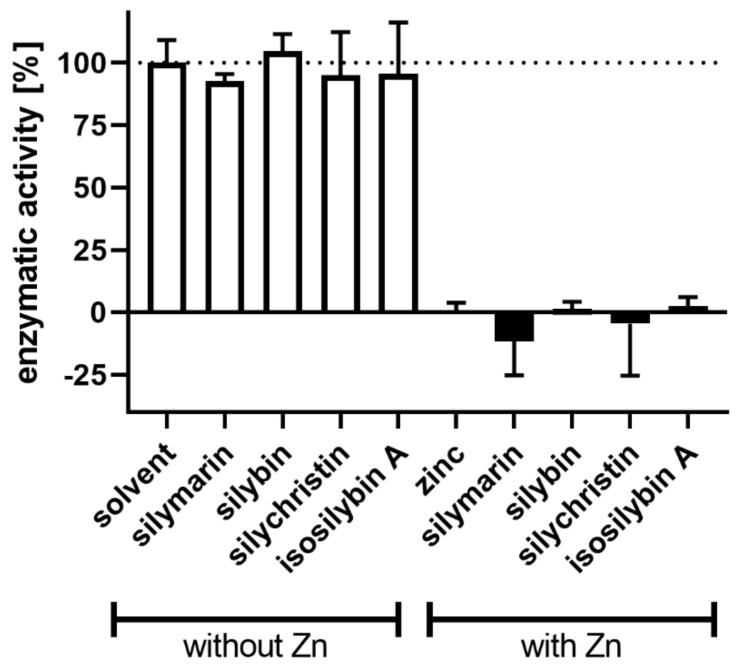
Effect of silymarin and flavonolignans on bovine glutamate dehydrogenase. The data reports non-significant effects of silymarin and flavonolignans on bovine glutamate dehydrogenase both tested alone or in combination with zinc ions. The final concentration of flavonolignans was always 50 µM while that of zinc 150 µM. The percentage is always related to the control, in the case without zinc to the enzyme-treated only with the solvent (DMSO), in case of zinc to the enzyme inhibited by zinc ions.

To gain better insight into the interactions between zinc ions and the flavonolignans, we chose a different enzyme, bovine glutamate dehydrogenase, which is known to be inactivated by zinc ions [29]. Indeed, increasing concentrations of zinc ions completely blocked the enzymatic activity of this enzyme (Figure 19). We chose a zinc concentration of 150 µM that partially blocked the enzyme, to observe possible recovery or further inhibition of the enzyme. As expected, the standard zinc chelator TPEN completely reversed the blockade by zinc and did not affect the enzyme in the absence of zinc (Figure 20). In contrast, both 2,3-dehydroflavonolignans were unable to restore the enzymatic activity. The reason for this was probably the fact that they were particularly potent inhibitors of the enzyme itself. Moreover, their inhibitory effect increased in the presence of zinc ions. Silymarin or other non-chelating flavonolignans had no effect on the enzyme both in the presence and absence of zinc ions (Figure 21).

## 4. Discussion

Silymarin is traditionally used for many different liver disorders. Despite its widespread use, clinical evidence is rather sparse, except for its potential application in the management of *Amanita phalloides* poisoning [30]. In about 150 documented cases, the overall mortality in patients treated with silymarin was about 8%, compared with more than 15% in cases not treated with silymarin [31]. Beneficial effects of silymarin on alcohol-induced toxicity have also been suggested [32], but it is unclear whether the inhibition of ADH might contribute to this effect.

Most of the toxic effects of ethanol are not caused by ethanol itself, but by its toxic metabolites, particularly acetaldehyde [33]. Acetaldehyde produced by the oxidation of ethanol is more reactive than ethanol itself and it promotes the production of reactive oxygen species (ROS) and also affects transcriptional activity [34]. The rate of ethanol conversion may therefore be critical to its toxicity. By inhibiting, or at least slowing down this metabolic pathway, the generation of acetaldehyde can be attenuated with subsequent amelioration of the oxidative stress caused by acetaldehyde and ROS. The microsomal ethanol oxidizing system, consisting primarily of CYP 450 2E1, has a lower catalytic efficiency than ADH and contributes to approximately 10% of ethanol metabolism. It is, however, inducible and it must be mentioned that it is responsible for faster alcohol clearance in chronic drinkers [11]. Regardless, in this study, we tested Zn-containing ADH, which is the major contributor to acetaldehyde formation and a crucial player in alcohol first-pass metabolism. There are several isoenzymes present in the stomach with different Michaelis constants *K*_M_ for ethanol, and their relevance is therefore dependent on alcohol intake. The metabolic capacity of these enzymes is also dependent on various factors including sex, age, and chronic alcohol consumption [10,11].

The effect of silymarin components on ADH has only been investigated in two ex vivo studies in rats. Interestingly, neither oral silymarin nor intravenous silybin hemisuccinate were able to affect rat liver ADH activity [35,36]. In this study, we first aimed to test the in vitro effects of purified silymarin flavonolignans and their metabolites on two different ADH enzymes to provide a solid basis for a possible in vivo study. As far as we know, the direct effects of pure silymarin flavonolignans and their sulfate conjugates on the enzymatic activity of ADH have never been tested before. In our setting, the tested compounds exhibited inhibitory activity against yeast and equine alcohol dehydrogenase, with higher activity on the equine enzyme overall. Interestingly, we found an enormous difference in the inhibition of the yeast and equine enzyme with the standard inhibitor fomepizole. Its IC_50_ for the yeast enzyme is almost 14 mM, while approximately 5 μM is needed for equine ADH. However, this is consistent with the literature, where the inhibitory effect of fomepizole (measured as *Ki*) on different bacterial ADHs ranged from 0.5 mM to 18 mM [37], while with the equine enzyme it was 80 nM [38] and with the human enzyme, it ranged from 0.1 to 2.1 μM [39,40,41]. The mechanism of action of pyrazole and 4-methylpyrazole (fomepizole) was formerly described as the formation of an inactive strong ternary complex by occupying the ethanol binding sites of the enzymes [42], however, it was also hypothesized that 4-methylpyrazole acts by coordinating the catalytic Zn^2+^ ions [43]. The general subunit conformations and enzymatic mechanisms of the yeast and equine enzymes are largely identical [16,44]. The amino acid sequence differences are more pronounced in the catalytic domain of these enzymes than in the coenzyme binding domain, and many of these replacements are located on the surface [45]. The zinc ion is conserved, and indeed the effect of the experimental zinc chelator TPEN was comparable between both our assays, with an IC_5O_ of 1723 μM for equine ADH and 654 μM for yeast ADH. However, with equine ADH, the maximal inhibitory effect of TPEN was lower than that of fomepizole. This is in agreement with the work by Meussen et al., where TPEN was only able to displace about 30% of the zinc from ADH, primarily the structural zinc, not the catalytic one [46]. Since the amount of the enzyme was not the same in both assays, some difference is logical, but it has a relatively low magnitude compared to fomepizole. Considering the fact that these enzymes are reliant on zinc atoms for both catalytic and structural purposes, zinc chelation can logically block or at least impede the enzymatic activity. Therefore, we examined the ability of a series of pure silymarin flavonolignans and some of their sulfated metabolites. Some of these compounds have already been shown to chelate copper and iron [15]. The structural requirements include a chelating site between hydroxy-hydroxy or hydroxy-keto groups and indeed in this study, the most active compounds were 2,3-dehydroflavonolignans including a hydroxy-keto moiety. The chelation mechanism also seems to be supported by the fact that the stereoisomers of 2,3-dehydrosilybin have almost the same activity. The different effect of 2,3-dehydrosilybin and 2,3-dehydrosilychristin on the two enzymes and on the zinc chelation might be due to the presence of an additional hydroxygroup at C-15 in the structure of 2,3-dehydrosilychristin (Figure 1).

There exist only scarce data on zinc chelation by flavonolignans in the literature. In one study, the synthesis of a complex of silybin and Zn^2+^ was carried out and the structure of the complex was proposed. The carbonyl group at C-4 was found to be crucial for complex formation, but it was not determined whether the second binding group was a hydroxy group at the C-3 or C-5 position [47]. More data are available on flavonoids that are structurally related to flavonolignans. Since the chelating sites of both flavonoids and flavonolignans are similar, we can effectively compare them [48,49]. The main chelation site of compounds with a flavonoid core is between the C-3-hydroxy and C-4-carbonyl groups. However, there is a significant difference between the presence or absence of the C-2,3 double bond. C-2,3-saturated structures (in our study silybin A and B, silychristin, and isosilybin A) exhibited no or a low ability to bind zinc ions. The presence of delocalized electrons between C-2, C-3, and ring B in combination with planar geometry between the hydroxy group on C-3 and the keto group on C-4 seems to be essential for the chelating ability of flavonolignans. A similar structure-effect relationship was observed when measuring the interaction of flavonolignans with Cu^2+^ and Fe^2/3+^ ions [15]. However, differences have been observed between 2,3-dehydroflavonolignans (2,3-dehydrosilybin and 2,3-dehydrosilychristin), which have the same flavonoid part of the molecule and thus contain the above-mentioned 3-hydroxy-4-keto chelating site. It is hence likely that the hydroxy group in ring B can influence the chelation. A similar phenomenon, i.e., a significant difference in cupric chelation was observed between kaempferol and morin [50], which differ only in the presence of a hydroxy group at the position C-2′ of ring B. Nevertheless, another chelating site exists between C-5-hydroxy and C-4-keto groups [48], but apparently, the presence of this moiety in flavonolignans is not associated with any strong chelation activity of Zn^2+^ ions. A chelating site between two molecules with C-7-OH groups on ring A was also described [49], but this chelation site is somewhat controversial.

In any case, the inhibition of ADH by 2,3-dehydrosilybin cannot be explained by zinc chelation alone. This largely stems from the fact that the potent zinc chelator TPEN was a less active inhibitor of equine ADH than 2,3-dehydrosilybin. Given the small area of the Zn^2+^-containing active center of ADH, this phenomenon can be explained by the inaccessibility of the large TPEN molecule to this site. In contrast, our molecular modelling revealed significant binding of three 2,3-dehydroflavonolignans in the substrate area near the nicotinamide moiety of NAD^+^/NADH and in proximity to the catalytic zinc atom and thus could explain their potential inhibitory potential and support our experimental results. Indeed, additional experiments have shown that both 2,3-dehydroflavonolignans blocked glutamate dehydrogenase activity at relatively low concentrations and that their effect was rather potentiated by zinc ions, in contrast to the standard zinc chelator TPEN. These results undermine the potential of 2,3-dehydroflavonolignans to modulate the enzymatic activity by zinc chelation. On the other hand, these compounds are less effective zinc chelators than TPEN, and their complexes may not reverse the inhibitory potential of zinc. Moreover, the inhibition of the enzyme appears to be particularly potent and can outweigh the possible interference with Zn. The fact that 2,3-dehydroflavonolignans are inhibitors of glutamate dehydrogenase is a new and unexpected finding. It is known that this enzyme is tightly and complexly regulated [51]. Previous studies have shown that the flavanol epicatechin does not affect it, but its gallate derivatives, which have an additional ring, blocked it strongly [52]. The flavonolignans tested in this study also contain additional rings compared to flavonoids. However, structural aspects, e.g., the binding site of epigallocatechin gallates, are not known, so the possible analogy in inhibition cannot be assessed at present.

## 5. Conclusions

This study shows that 2,3-dehydroflavonolignans from silymarin are able to moderately chelate zinc. 2,3-Dehydrosilybin was a much stronger chelator of zinc than 2,3-dehydrosilychristin under neutral conditions. Both compounds partially inhibited yeast ADH, and 2,3-dehydrosilybin had a more pronounced effect on equine ADH, a relevant model of human ADH. These results were confirmed by in silico docking. This observed inhibition of ADH by 2,3-dehydrosilybin may have some real biological consequences. On the one hand, the ability to affect ADH in the liver is unlikely due to the low bioavailability of flavonolignans [53]. Although the pharmacokinetics of 2,3-dehydroflavonolignans are not well described, their systemic absorption is similar to that of other flavonolignans, at least in rabbits [54]. On the other hand, a local effect in the gastrointestinal tract on gastric ADH is possible and more likely than a systemic one. The fact that it was observed at relatively low concentrations of 10 µM supports its possible biological relevance.

The main advantages of this study are that we tested pure flavonolignans from silymarin and not the mixture itself, and compared their zinc-chelating activity as well as their effect on ADH. It should be also emphasized that this study has several limits, in particular the use of yeast/horse ADHs instead of different human ADH isoforms, and the absence of confirmation of these results on animals. Hence in the future, the biological relevance of our findings must be confirmed as well on the whole organism level.
